# Public and mental health professionals’ perspectives on social media and suicide exposure

**DOI:** 10.1186/s12889-025-22587-6

**Published:** 2025-04-12

**Authors:** Jo Bell, Christopher Westoby

**Affiliations:** 1 School of Psychology and Social Work, University of Hull, Hull, UK; 2 School of Humanities, University of Hull, Hull, UK

**Keywords:** Suicide prevention, Social media, Exposure, Public health, Mental health, Postvention, Communities, Impact

## Abstract

**Background:**

The rapid evolution of social media in recent years has increased public exposure to suicide. While research has highlighted concerns about the role of social media in facilitating harmful discourse and imitative suicidal behaviour, there is a wide gap in our understanding of the impact of social media exposure in the aftermath of a suicide, and no research to our knowledge from preventive public and mental health perspectives. This qualitative study explored the experiences of public and mental health professionals in relation to social media exposure following a suicide. The study aimed to (1) Better understand social media risks from a public health perspective; (2) Provide insights for public health policy and strategy to enhance suicide prevention efforts and inform guidance for responding.

**Methods:**

In-depth interviews were conducted with 10 purposively sampled public and mental health professionals based on their roles in crisis response, suicide prevention, and digital monitoring. Data were collected through semi-structured interviews, focusing on their experiences of responding to suicide-related risks on social media, including the spread of information across platforms, public engagement with content, impact, and intervention challenges. Data were analysed using thematic analysis to identify emerging themes.

**Results:**

Four key themes emerged: (1) The communicative ecology of social media (where the public act as content purveyors, rapidly disseminating varied and often unregulated narratives); (2) Harmful effects (including the copycat effect and toxic discourse); (3) Positive effects (where protective discourse and moderation offer harm reduction opportunities); and (4) Challenges in intervention (including content moderation difficulties and algorithmic biases that amplify harmful narratives).

**Conclusion:**

Findings highlight the need for improved content moderation, public health-led digital monitoring, and education on safe social media use. Strengthening real-time suicide surveillance, improving collaboration with social media platforms, and promoting public awareness of digital literacy are critical to mitigating risks and ensuring social media supports suicide prevention efforts. As digital communication continues to evolve, proactive public health strategies will be essential in safeguarding vulnerable individuals.

## Background

Suicide is a serious global public health problem that demands attention. Each year between 700,000 and 800,000 people die by suicide globally [1]. In England and Wales estimates are between 5000 and 6000 deaths per year [2]. From a public health perspective, suicide prevention involves a wide range of activities that varies from provision of optimal conditions for healthy growth and development, to timely support and treatment for mental health conditions, to the restriction of the means of suicide and more [1]. Appropriate dissemination of information, provision of resources and awareness-raising are essential elements in successful suicide prevention. Responsible media reporting about suicide is also a critical component. It is now widely accepted that exposure to suicide via media can play a vital role in shaping public understanding of suicide and can influence actual behaviours. The effect of media coverage on ‘copycat’ suicides and suicidal behaviour (‘Werther effect’ [3]) has been recognised as an important public health issue for at least 50 years. Phillips [3] used the term to describe suicides that occur as a result of exposure to media reporting of another suicide [4]. In essence, the greater (more repetitive or wide-reaching) the coverage of a suicide story, the greater the chances of finding a ‘copycat’ or ‘contagion’ effect [5]. Recent research also highlights the ‘Papageno effect’ [6] demonstrating that under certain conditions, representations of suicide can lower the risk of imitative suicides and have a positive preventive effect.

However, traditional media now exists alongside and within an instantaneous sharing of information (social media) that can be created and controlled by anyone and concerns about the impact of exposure have been heightened since [7], particularly given the speed at which unregulated content and potentially distressing content about suicide can spread.

News about a suicide can spread rapidly across different digital platforms and be interacted with multiple times by users, contributing to the narratives and often fuelling speculation and different versions of events [8, 9]. Exposure to suicide-related content has therefore become far more complex than ever before and the number of people exposed in this way has multiplied exponentially. Bell and Westoby [8, 9] examined this through the lens of polymedia which revealed the potential impact and scope of social media exposure following a suicide, including the potential for increased risk of contagion. They argued that media guidelines on safe reporting must be updated to account for the globalisation of new media technologies.

A number of studies have examined the impact of social media on actual (copycat) suicides and, consistent with research findings for traditional media, exposure to graphic and distressing suicide-related content on social media has been linked to an increase in suicidal thoughts and behaviours [10]. A recent systematic review by Calvo et al. (2024) [11] identified 25 studies which examined whether the Werther effect occurs in response to social media across nine different countries, and distinguished at least seven different types of social media. The review concluded that social media can act as a source of suicidal contagion but also that there is evidence of a protective effect. They also found that the complexity of the dissemination of suicide-related content is higher than expected, suggesting that what matters most is the context and characteristics of the exposition (a point also noted by Bell and Westoby [8, 9] above). One of the most significant limitations found, however, was the heterogeneity of the articles (with differences in social media types, content examined, designs employed and samples and measures used) making comparisons and generalisations of results problematic. Thus, while recent research has examined the harmful impact of exposure to suicide-related content, including some which have studied specific social media platforms (such as Instagram [12–15], Pinterest [16] and Twitter [15, 17, 18]) this field of research is still underexplored. Studies such as those by Umrah-Dawes et al. [15] and Picardo et al. [14] emphasise the need for further in-depth understanding through research using direct interviews with users. Additionally, research by Arendt [12], Carlyle et al. [13], and Guidry et al. [16] has called for greater involvement of public health and mental health professionals in applying media guidelines, again highlighting the challenges posed by guidelines designed in a pre-digital era.

Guidelines for responsible media reporting of suicide stress the importance of avoiding, sensationalizing, glamorizing, or normalizing suicidal behaviour, and instead promoting positive mental health stories and local support resources. Recent updates to WHO guidelines [1] include, for the first time, new recommendations and specific considerations for online, digital and social media. However, these guidelines are primarily tailored for media professionals and do not adequately address the role of the broader public, who can now act as producers and purveyors of suicide-related content on social media. While some important efforts have been made in the development and testing of guidelines for young people on how to communicate safely about suicide online (e.g., #chatsafe) [19, 20] with promising results, there is still a lack of evidence on the role and impact of social media exposure and these considerations have yet to filter into public health policy, guidance and practice.

As the general public generates and interacts with content related to suicides on social media, the task of mitigating the risks posed has become a fundamental challenge for both suicide prevention and public health policy. This is important because public and mental health providers (including those who work in postvention, public health, police, and mental health services) play a vital role in responding in the aftermath of a suicide. This work often takes place ‘behind the scenes’, ensuring public safely and minimising exposure. While many within these services and organisations are aware of social media’s influence (as noted by Bell and Westoby [8]), there is a lack of clear guidance to address this complex issue.

This paper reports on a qualitative study involving in-depth interviews with public and mental health providers who respond in the aftermath of a suspected suicide [21]. By focusing on their experiences and observations, the study aimed to:

1. Better understand social media risks from a public health perspective.
2. Provide insights for public health policy and practice to enhance suicide prevention efforts and inform guidance for responding to social media exposure following a suicide.

## Methods

In-depth interviews were undertaken with a purposive sample of participants working in public health and mental health services with a role in responding following a suicide. The sample was drawn from a wider pool of people who attended a Knowledge Exchange workshop, which invited individuals working in public services supporting those affected by suicide, or who had a professional and/or personal involvement in suicide prevention work. The aim of the workshops was twofold. Firstly, to bring together a range of perspectives to educate and share knowledge on social media use following a suicide and impact on communities. Secondly, to gather further insight into professionals’ experiences to improve future responses regarding social media.

Five workshops were delivered in four different locations in England. They were developed and delivered by the authors, alongside a suicide prevention expert (responsible for the implementation of regional suicide prevention strategy) and an expert by experience. Workshops lasted for half a day, beginning with an educational element (covering content pertaining to the uniqueness of suicide bereavement, and the harmful and protective effects of social media use after a suicide). Attendees were then invited to discuss their observations and experiences of social media use following a suicide and its impact from the perspective of their own professions. Detailed notes were taken by one facilitator and used to help develop topic guides for subsequent semi-structured in-depth interviews. Following the workshops, attendees were invited to participate in a research interview. A purposive sample was then drawn from this list based on participants’ roles in crisis response, suicide prevention, and digital monitoring.

### Interviews

The study received ethical approval from the Faculty of Health Sciences Research Ethics Committee at the University of Hull, UK. Participants consented to be recorded, and for their anonymised data to be used in publications. All participants were provided with contact information for relevant support resources.

A semi-structured interview guide featuring open-ended questions was used. Question topics covered participants’ experiences and observations of: polymediated exposure to suicide (including how social media operates and the context within which it is used), the complexities of digital media communication, activities on various social media platforms, protective and harmful effects on individuals and communities, and challenges for intervention and prevention in public health.

### Data analysis

Interview data were analysed using a thematic analysis approach [22], incorporating the constant comparative method [23]. This approach allowed us to identify patterns, themes, and variations in participants’ experiences while ensuring an iterative and reflexive coding process.

All interviews were audio-recorded and transcribed verbatim. Any unclear sections were clarified through repeated listening. The research team (authors) read and re-read the transcripts to gain a deep understanding of the data and to begin identifying recurring ideas or key points. Initial notes and memos were taken to document early impressions, patterns, and significant observations. An open coding process was then used to identify key concepts within the data. Segments of text were assigned codes based on emerging themes. The constant comparative method was then applied, meaning each new code was compared with existing codes to ensure consistency and refinement. To develop and refine themes, codes were grouped into broader themes based on conceptual similarities. Themes were reviewed, ensuring they accurately represented the data and were not redundant or overly broad and any new codes that emerged prompted a re-evaluation of earlier coded data to ensure comprehensiveness. The research team engaged in reflexive discussion and consensus-building to validate themes and ensure credibility. Finally, themes were organised into a coherent structure, highlighting key findings relevant to public health responses. Written narratives were constructed around each theme, incorporating illustrative quotes from participants to ground the findings in real-world experiences.

## Results

A total of 67 people attended the five workshops. Ten people were purposively sampled to be interviewed from this group on the basis of their knowledge and experience; one participant was recruited via snowballing after hearing about the research at a public webinar delivered by the authors. Interviews lasted approximately one hour. Table 1 details all participants, whose names have been pseudonymised.

**Table 1:** Interviewees

Colleen	Public Health Lead (suicide prevention remit)
Milly	Public Health Lead (suicide prevention remit)
Paul	Public Health Media Professional (suicide prevention remit)
Emma	Mental Health Support Worker (university setting)
Verity	Mental Health Support Worker (university setting)
Heather	Dep Chief Operating Officer (suicide prevention in public places)
Tilly	Adolescent Mental Health Senior Support Worker (inpatient unit)
Christine	Social Worker (homeless mental health team)
Phoebe	Healthcare Assistant (homeless mental health team)
Barry	Social Housing Officer (mental health)
Oliver	Police Inspector (suicide prevention remit)

Four main themes (with sub-themes) were developed from the analysis: (1) Communicative ecology of social media (public as purveyors, multiple narratives); (2) Harmful effects on individuals and communities (copycat effect, toxic discourse); (3) Positive effects on individuals and communities (protective discourse); (4) Challenges and opportunities for intervention and prevention (content moderation, algorithmic bias). These are presented in turn below.

### The communicative ecology of social media

All participants (see Table 1) observed the polymediated transmission of suicide-related content on social media, reflecting on the evolving network of communication processes, participants, and technologies that shape digital information flow. The increasing interconnectivity between platforms allows content to spread rapidly across multiple channels, often without clear oversight. Algorithms play a central role in curating exposure, amplifying content based on user engagement, preferences, and trends. As a result, individuals—whether directly affected or not—can become part of a wider discourse surrounding suicide, contributing to its dissemination and shaping public narratives.

### Public as purveyors

Participants described how ordinary social media users, rather than traditional media sources, often play a key role in spreading information about suicide. Tilly reported that young people in her care had been exposed to explicit details of suicide methods and locations through spiralling engagement on TikTok. Videos, sometimes created by adolescents in inpatient units, circulated widely, including instructions on how to avoid detection (Tilly, 203).

Emma described how suicide-related stories originating from publicly visible incidents were rapidly shared on community platforms, fuelling widespread discussion. In one instance, a bystander posted live updates of a suicide attempt in a public place, which was instantly disseminated. In another, what began as a police report of an “incident” on Facebook was quickly reposted across local community groups and spread further via WhatsApp (259–295):

[My sister’s] *friend had screenshotted it and sent it to her… My sister sent it to me and my mum… We don’t know anybody involved in that situation and somehow it’s in my family WhatsApp group chat… I’m not even on Facebook and I’ve seen a Facebook post that someone else has posted in a group.* (282)

Barry described similar incidents in his area, where members of the public photographed suicide scenes and uploaded them to Twitter and Reddit, making distressing images widely accessible (Barry, 665).

These examples illustrate how social media blurs the boundary between private and public information, allowing sensitive content to spread rapidly without formal verification or ethical considerations.

### Multiple narratives

Participants also highlighted how social media amplifies fragmented and evolving narratives of suicide incidents, often shaped by both journalists and the public. Heather and Oliver observed that local news media actively search social media for emerging stories, sometimes reposting unverified content (Heather, 59; Oliver, 78).

Heather described a case in which a suicide in a public place became a rolling news story, repeatedly updated and reshared:

*Every time there was an update… they just regurgitate it*,* over*,* over and over again.* (354)

This constant stream of information fuelled speculation, including misidentification of the individual involved. Such cases underscore how the interconnected, real-time nature of digital platforms allows suicide stories to take on a life of their own, with details evolving through public engagement and resharing.

### Negative impact of social media exposure

All participants highlighted the adverse consequences of social media exposure following a suicide, particularly its role in facilitating imitative behaviour and amplifying toxic discourse. These dynamics contributed to increased risk for vulnerable individuals and posed significant challenges for public health professionals in managing community responses.

### ‘Copycat’ effect

Several participants emphasised how publicly disclosing the location of suicides on social media could trigger imitative behaviours. Colleen shared an example from her professional experience in which a bystander intervened to prevent a suicide attempt in a highly visible public area but later shared the exact location on social media (182–194). In the following two days, suicide attempts at the location increased fivefold, prompting concerns about the role of social media in reinforcing suicidal behaviour:

*We had eleven attempts in two nights… Typically*,* we have one per night.* (537)

In another case, Colleen recounted a suicide attempt at a prominent city-centre building that attracted significant media coverage, including disclosure of the location. In the days following, multiple individuals in crisis visited the site, ultimately requiring round-the-clock security (212–222).

Similarly, Heather observed a “significant increase” in distressed individuals gathering at another public location following online discussions that disclosed a recent suicide site (133–164). These incidents underscore the public health risk posed by location-sharing, reinforcing the need for policies that restrict the disclosure of suicide sites to minimise the potential for contagion.

### Toxic discourse

Participants also described the harmful impact of toxic online discussions following suicides. Phoebe, whose organisation had experienced a series of suicides in quick succession, recalled the “absolutely awful” nature of online comments, estimating that “75%” were judgmental or dismissive (103–116).

Barry provided an example of toxic Facebook discussions where community members trivialised a suicide, with some even making jokes (460). He noted that conversations often turned “quite nasty”, distorting the narrative around the deceased and leading their sibling to monitor social media in an attempt to remove distressing content (501, 514).

Christine and Phoebe further observed how social media discussions blamed mental health services for perceived failures, with online narratives portraying a lack of meaningful support for those in crisis. Phoebe described the situation as “horrendous”, emphasising how blaming services became a recurring theme in local Facebook groups and online news media (94). Christine speculated that such discourse could discourage vulnerable individuals from seeking help, reinforcing a sense of hopelessness (642). The prevailing narrative conveyed the notion that suicide is a deplorable, tragic act, but there is a lack of meaningful help available for those who feel this way.

### Positive impact of social media exposure

While participants highlighted the risks associated with social media exposure following a suicide, they also acknowledged its potential for harm reduction and support, particularly through protective discourse and community-driven interventions.

*The flipside of it is… there’s a lot of good stuff that gets shared as well… It’s never been easier to share advice*,* it’s never been easier to share helplines.* (Paul, 361)

Participants described efforts to mitigate harm by promoting protective messaging and challenging harmful content online. Paul shared an example in which he observed distressing social media activity following the death of a young person. Recognising the potential risks, he alerted the local suicide prevention network, enabling them to intervene before the situation escalated (486–493).

Milly highlighted a community-led initiative where “pro-social messages” were disseminated on social media following a suicide. These messages included guidance on accessing help, addressing loneliness, and signposting services, leading to increased referrals and engagement with local mental health resources (918).

Tilly shared a case where a young person died by using a method that had gained visibility on TikTok. In response, the individual’s parent used the same platform to share protective content with the same audience, aiming to counteract harmful exposure with helpful information (498–501).

Heather also observed a growing culture of empathy and openness among young people on social media. She noted an increase in kindness and understanding toward those affected by suicide, offering a sense of hope:

*They seemed to understand that they could experience mental health challenges*,* recover from them*,* and openly discuss them.* (393–398)

Despite these positive developments, participants expressed concern over the visibility and impact of protective discourse. Colleen noted the difficulty in assessing the effectiveness of harm reduction efforts, explaining that:

*Those victories are quite silent when the failures are much… louder and more public.* (554–560)

Similarly, Phoebe observed that recovery stories and positive narratives were often overshadowed by negative content, particularly critical discussions about mental health service failures. She speculated that, although hopeful stories exist, they are less likely to gain traction because they receive fewer interactions, likes, and comments, making them less visible due to social media algorithms (681).

### Challenges and opportunities for intervention

Participants reflected on the challenges and opportunities for public health intervention on social media, emphasising content moderation difficulties and algorithmic bias. The findings highlight platform-specific obstacles, the spread of information beyond institutional control, and the role of algorithms in shaping user engagement with suicide-related content.

### Content moderation

Participants described significant barriers to moderating content across different platforms, including anonymity, ephemeral messaging, and the rapid interconnectivity between digital spaces. Tilly highlighted TikTok’s lack of user verification, making accountability difficult:

*Nobody knows who you are.* (635)

In the context of the spread of news within a university setting, Emma and Verity noted that news of a suicide often spreads across multiple platforms (e.g., WhatsApp, Facebook, Twitter, Instagram, Snapchat, TikTok) before official announcements can be made, limiting the ability of public health teams to control initial responses or provide immediate support:

*We have so many things that we could do in the aftermath*,* but the reality is we would not be breaking the news to anybody… That’s difficult because then you’re not in control of supporting the initial aftershocks.* (Emma, 339–372)

Milly shared her experience managing a suicide cluster affecting young people, where Snapchat played a key role in exposing users to suicide-related content. She emphasised the ephemeral nature of Snapchat’s sharing of content and its restricted access, which hindered monitoring and intervention efforts:

*We couldn’t possibly pick out all of the young people that might be affected… With Snapchat… things disappear after twenty-four hours and you’ve got to be friends with somebody to see them*. (Milly, 218–229)

She compared Snapchat, Facebook, and WhatsApp, noting that while Facebook allows some degree of public monitoring, Snapchat and WhatsApp function in more private spheres, making intervention nearly impossible without insider access (230–247).

Colleen provided an example of the extensive time required to remove concerning content, recounting a case where a young person who later died by suicide had previously posted on Twitter. The post remained online for months, despite concerns about its potential for suicide contagion (164).

### Algorithmic Bias

Participants reflected on the influence of social media algorithms in amplifying harmful narratives. Tilly discussed TikTok’s algorithm-driven content curation, explaining how users engaging with suicide-related content (even passively) are drawn into “spirals” of similar content through hashtag-driven recommendations (193, 210–235). This means that users may inadvertently be exposed to more suicide-related material, increasing potential risks.

Phoebe, Emma, Varity and Colleen questioned whether protective content is systematically deprioritised, as algorithms push the most interacted-with comments to the top—which are often sensationalist, negative or harmful—while supportive comments remain buried:

*In order for protective content to be seen enough to make an impact*,* it needs algorithms on its side.* (Phoebe, 703–704)

## Discussion

Findings from our study highlight challenges for public health professionals in mitigating social media risks, particularly as exposure to explicit content and speculation can exacerbate distress within communities. The ease with which multi-perspective narratives emerge, often unregulated and shaped by public participation, raises concerns about how best to intervene without infringing on public discourse. Participants emphasised that disclosure of suicide methods and locations can contribute to copycat behaviours, reinforcing previous findings that location-sharing can drive imitative acts. At the same time, our study illustrates how toxic discourse—marked by hostility, misinformation and blame can undermine constructive dialogue. These findings underscore the urgent need for public health interventions to address toxic discussions online and strengthen content moderation strategies to prevent harmful narratives from shaping public perceptions of suicide and mental health services.

Despite these risks, our study also highlights the potential of protective discourse—messaging that fosters hope, recovery, and well-being. Public health and mental health professionals are actively engaged in monitoring, removing unsafe content, and promoting protective messaging to counteract the Werther effect. These efforts align with previous research, which suggests that social media can be a valuable tool for suicide prevention if strategically used to amplify protective narratives. However, our findings suggest that protective content may struggle for visibility, particularly in platforms where engagement-driven algorithms elevate harmful discourse over supportive messages.

This challenge is compounded by platform-specific barriers to intervention. Findings illustrate the limited ability to monitor certain platforms, particularly in private group settings and those where content is ephemeral. Even on platforms where content is more accessible, delayed content removal can prolong exposure to harmful narratives. Similarly, lack of user accountability can make it difficult to trace the sources of harmful content. These challenges highlight the need for stronger collaboration between public health professionals, social media platforms, and policymakers to improve monitoring and intervention efforts.

Our findings align with previous research [16] indicating that negative comments attract more responses than positive ones. This, in turn, aligns with broader concerns about social media’s role in spreading low-credibility content [24], which poses risks in other public health contexts [25, 26]. However, as others have noted [18] enforcing moderation and regulation of online content remains extremely challenging, as intervention efforts are often delayed, difficult to enforce, and constrained by platform-specific limitations. Our findings suggest these challenges may be further compounded in the context of suicide-related content, where moderation efforts may compete with algorithms that inadvertently amplify the Werther effect.

These findings raise critical questions about the adequacy of existing media guidelines in a digitally driven media landscape (as highlighted by previous research [8, 9, 16, 17]). While traditional media has long been subject to suicide reporting guidelines, social media operates within a fragmented regulatory environment where content is shaped by user interactions rather than editorial oversight. Our study underscores the need for proactive policy measures that prioritise the visibility of protective content, improve the responsiveness of moderation systems, and facilitate greater transparency in how platforms handle suicide-related discourse.

Our findings offer insights for new policy and practice approaches in public health. To mitigate the risks associated with social media exposure following a suicide, public health efforts must focus on enhancing preparedness, strengthening platform accountability, and fostering responsible digital engagement. A key strategy involves incorporating social media monitoring into Real-Time Suicide Surveillance (RTSS) systems to track suicide-related discourse and digital exposure as it unfolds. RTSS, which collects and analyses trends in suicidal behaviour, could provide public health professionals with timely insights into emerging risks, allowing them to intervene swiftly to prevent contagion and support at-risk individuals [27]. By integrating social media exposure data into RTSS, public health teams could develop targeted interventions that curb toxic discourse, misinformation, and harmful narratives while actively promoting protective content that directs users to reliable resources and encourages constructive dialogue.

Beyond public health monitoring, social media companies could take greater responsibility for ensuring that harmful content is minimised and that their platforms promote accuracy, fairness, and ethical discourse. Addressing algorithmic bias is central to this effort, as content recommendation systems play a significant role in shaping the visibility of suicide-related discussions. A coordinated effort among platforms [28] could lead to more effective strategies for amplifying protective content while reducing exposure to harmful narratives. Improving content moderation policies and removal processes would allow for faster intervention when high-risk content is identified, while streamlining user reporting mechanisms would empower individuals to flag harmful material more easily.

Public health efforts must also recognise the importance of social norms in shaping online discourse. A shift toward more responsible and informed engagement with suicide-related content is needed. Education and awareness campaigns could focus on promoting digital media literacy, encouraging users to critically evaluate content rather than relying on popularity metrics such as likes and shares. Equally important is fostering a culture of thoughtful engagement and respectful, constructive discourse, where users pause before posting or responding to sensitive material, to consider its potential impact. To improve discourse across social media, broader cultural change is needed to establish clearer norms for discussing suicide in ways that are respectful, sensitive and aligned with the principles of the Papageno effect.

### Limitations and positionality

The study was based on in-depth interviews with public health and mental health professionals. While their perspectives offer rich detail, they may not fully represent the range of experiences across different regions, roles, or institutional settings. Given participants’ professional focus on crisis response and mitigation, discussions may have emphasised risks and harms more than protective aspects of social media. While some benefits were acknowledged, they may have been minimised or made less visible in the data.

The number of interviews is lower than the conventional threshold for qualitative research. However, the interview topics were grounded in themes emerging from the workshop discussions with the broader group of 67 professionals. This approach ensured that the interviews were informed by a wider practitioner experience base, rather than being developed in isolation. We recognise that further research with a larger sample would be beneficial to confirm and extend our findings.

As researchers, we recognise that our positionality influenced the study, shaping our approach to data collection, analysis, and interpretation. Our backgrounds in public and mental health, and digital media studies provided a lens through which we examined these issues, but also introduced certain biases. While our expertise informed the inquiry, we remained committed to reflexivity—actively questioning our assumptions and emerging findings to enhance credibility. Our prior knowledge of the topic may have shaped how we framed participants’ narratives, despite efforts to centre their lived experiences; we acknowledge that alternative interpretations may emerge from different disciplinary perspectives.

The study relied on qualitative, experience-based accounts rather than direct measurement of social media impact. While participants provided valuable insights based on professional judgment, there were inherent limitations in assessing the actual influence of social media exposure on affected communities. Despite these limitations, this study highlights critical challenges and opportunities for public health responses to social media following a suspected suicide. By explicitly acknowledging our positionality, we aim to enhance the trustworthiness of our findings and provide transparency regarding the perspectives that shaped this research.

## Conclusion

This study explored the observations and experiences of public health and mental health professionals in relation to social media exposure following a suspected suicide, with the aim of better understanding social media risks and benefits and informing public health strategies for intervention and prevention. Our findings highlight the complexities of digital communication, the potential for both harm and protection, and the challenges faced by professionals in monitoring, regulating, and responding to suicide-related discourse online.

Consistent with previous research, our results suggest that social media can both amplify suicide contagion risks and serve as a platform for harm reduction. The rapid circulation of misinformation, disclosure of methods and locations, and toxic discourse can contribute to contagion. Protective messaging offers the potential to mitigate some of these effects. However, protective content may struggle for visibility due to algorithmic bias, high engagement with negative content, and content moderation limitations across different platforms.

These findings reinforce the need for multi-layered public health responses that integrate real-time monitoring, stronger platform accountability, and public education on responsible digital engagement. Incorporating social media data into suicide surveillance systems could improve early intervention, while collaboration with platforms is necessary to mitigate harmful content amplification. Addressing these challenges requires cross-sector efforts to ensure that social media is not only safer but also actively contributes to suicide prevention efforts. As social media continues to evolve, proactive and adaptive public health strategies will be essential to ensure that online spaces are not only safer but also actively contribute to suicide prevention efforts.

## Data Availability

Data cannot be shared openly, to protect study participant privacy. Participants have not consented for their data to be shared. Data used in this study have been fully anonymised.
